# Phoenix and Fowl: Birds of a Feather

**DOI:** 10.3201/eid1111.AC1111

**Published:** 2005-11

**Authors:** Polyxeni Potter

**Affiliations:** * Centers for Disease Control and Prevention, Atlanta, Georgia, USA

**Keywords:** Art and science, emerging infectious diseases, Phoenix and Birds

**Figure:**
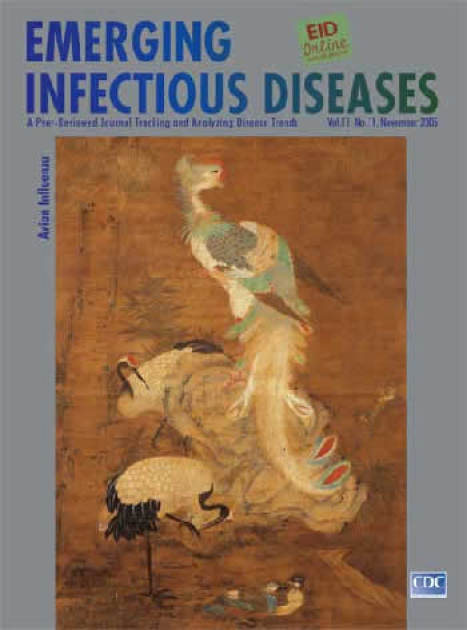
Phoenix and Birds. China (c. 16th century). Ink and colors on silk (213.4 cm × 113 cm). Honolulu Academy of Arts, Hawaii, USA. Gift of Charles M. and Anna C. Cooke Trust Fund, 1928 (141.1)

A treasured aviary, the vast collection of bird paintings in Chinese art reflects longstanding global fascination with our feathered friends. Balanced on two legs, like humans, and able to fly and swim, birds have been viewed as an engineering miracle in the East and West and have been studied by artists and scientists alike.

Traditional Chinese painting goes back 6,000 years to the Neolithic period and is found on early pottery decorated with brush images of humans and animals (*1*). Painting of flowers and birds originated on this primitive pottery, as well as on bronze and silk adorned with simple but brightly colored designs (*2*). The genre, which is seen throughout Chinese art history, flourished during the Song dynasty (1101–1125 AD).

During the Yuan dynasty (1279–1368), the Mongol wars and general turmoil under Genghis Khan overshadowed a strong artistic legacy enriched by diverse foreign influences. The period saw suspension of artists' and intellectuals' rights and retreat to traditional styles of painting (*3*). Need for greater artistic expression coincided with the return of native rule during the Ming dynasty.

The Ming dynasty (1368–1644) was a time of cultural restoration and expansion for the Chinese, a "scholar's culture" of thriving literary and artistic communities populated by writers, poets, and artists, many of them outstanding masters with extraordinary skills and breadth (*4*). Revived interest in local culture was often expressed in landscape images of mountains or other nature scenes painted on scrolls. Monochrome and color woodblock printing developed and advanced at this time, as did porcelain production and diversification. Yet, artists worked primarily in a revival of Song academic styles, prescribed by a conservative court for its glorification and prestige (*5*).

Genius is the most important quality in a painter, knowledge comes next, and "the single brush stroke is the source of all things," wrote painter and member of the Ming royal house, Shitao (1642–1707) (*6*). Unlike canvas, silk, which was used in painting even before paper was invented, was unforgiving of errors and required exceptional skill and confidence. Many renowned Chinese painters were also expert calligraphers and poets, who often made literary references in landscape painting, emphasizing the connection between disciplines and adding complexity to the work.

Phoenix and Birds, on this month's cover, exhibits many of the qualities of Ming dynasty silk scroll painting. The narrative content (The Five Human Relationships Represented by Five Different Birds) is expressed with surely executed lines and subtle colors in vertical format. Shadow, light, and proportion are used to create a third dimension. A central figure, the phoenix, dominates the scene. This legendary bird, part of global mythology, is described here in the Chinese tradition. Like the dragon, with which it is often associated, the phoenix, or *fenghuang*, exemplifies the union of yin and yang (polar opposites complementing each other in nature and underlying order within the universe). In some legends, the *fenghuang* is created from desirable parts of other creatures: cock's beak, swallow's face, fowl's forehead, snake's neck, goose's breast, tortoise's back, stag's hind, fish's tail. Its song reflects the notes of the musical scale, its feathers five fundamental colors, its figure the celestial bodies: head symbolizes the sky; eyes, the sun; back, the moon; feet, the earth; tail, the planets (*7*).

This emperor of birds is anchored on a rock, its royal plumes and fearless stare signaling preeminence. Below are two cranes, symbols of wisdom and longevity. They seem aware of their surroundings and of two other waterfowl fraternizing in the foreground.

In this harmonious bird scene, the unknown artist injects a measure of Confucian values, the need for each creature to act not singly but in connection with others, through five relationships: parent-child, husband-wife, sibling-sibling, friend-friend, ruler-subject, in networks of individual persons, the family, the state, the universe (*8*). This conglomeration of myth and Confucian wisdom within the Asian tradition has timeless implications. And in today's context, troubled by the specter of pandemic avian flu, Phoenix and Birds seems prophetic.

The bird ensemble captures issues at the heart of our current predicament: unknown pathogen origins, exotic composites of unlikely elements, increasing complexity, vast public health implications. The imperial phoenix with its patchwork beauty, perched high on the mount is not much different from the frolicking cranes or the humble fowl crouching anonymously in the foreground. All participate in nature's play. More than the sum of its unlikely parts, the phoenix recalls the flu virus and its wild recombination. Less conspicuously, the migrating waterfowl signal this species' importance as reservoir hosts and dissemination agents, bringing the virus to creatures absent from this painting (domestic poultry, swine). The circle is complete as new opportunities arise for recombination with local mammalian strains to form a new virus with pandemic potential. Confucian relationships meet nature's whim.
